# DeepAttNet: deep neural network incorporating cross-attention mechanism for subject-independent mental stress detection in passive brain–computer interfaces using bilateral ear-EEG

**DOI:** 10.3389/fnhum.2025.1685087

**Published:** 2025-11-03

**Authors:** Wooseok Hyung, Minsu Kim, Yesung Kim, Chang-Hwan Im

**Affiliations:** ^1^Department of Artificial Intelligence, Hanyang University, Seoul, Republic of Korea; ^2^Department of Electronic Engineering, Hanyang University, Seoul, Republic of Korea; ^3^Department of Biomedical Engineering, Hanyang University, Seoul, Republic of Korea

**Keywords:** electroencephalography (EEG), deep learning, ear-EEG, mental stress, passive brain–computer interface

## Abstract

**Introduction:**

Electroencephalography (EEG)-based mental stress detection has the potential to be applied in diverse real-world scenarios, including workplace safety, mental health monitoring, and human–computer interaction. However, most previous passive brain–computer interface (BCI) studies have employed EEG recorded during the performance of specific tasks, making the classification results susceptible to task engagement effects rather than reflecting stress alone. To address this limitation, we introduce a *rest-versus-rest* paradigm that compares resting EEG recorded immediately after exposure to a stressor with that recorded after meditation, thereby isolating mental stress from the task-related confounds. EEG recording setups were designed under the assumption of bilateral ear-EEG, a compact and discreet form factor suitable for real-world applications. Furthermore, we developed a novel subject-independent deep learning classifier tailored to model interhemispheric neural dynamics for enhanced mental stress detection performance.

**Methods:**

Thirty-two adults participated in the experiment. To classify mental stress status in a subject-independent manner, we proposed DeepAttNet, a deep learning model based on cross-attention and pointwise temporal compression, specifically designed to effectively capture left and right hemispherical interactions. Classification performance was assessed using eight-fold subject-level cross-validation against conventional deep learning models, including EEGNet, ShallowConvNet, DeepConvNet, and TSception. Ablation studies evaluated the impact of the cross-attention and/or pointwise compression modules.

**Results:**

DeepAttNet achieved the highest average accuracy and macro-F1 values, with performance declining when either the cross-attention or pointwise compression module was removed in the ablation studies. Explainability analyses indicated lower cross-attention entropy with stronger directional ear-to-ear asymmetry under stress, and temporal occlusion identified mid–late windows supporting stress decisions. Moreover, six of seven canonical scalp-EEG markers were FDR-significant for post-stressor vs. post-relaxation rest.

**Conclusion:**

The proposed rest-versus-rest paradigm and DeepAttNet enabled robust, subject-independent mental stress detection with a fairly high accuracy using only two-channel EEG recordings. This approach is expected to offer a practical solution for continuous stress monitoring, potentially advancing passive BCI applications outside laboratory settings.

## 1 Introduction

Mental stress is an adaptive response of the brain and body to perceived demands or pressure, briefly mobilizing energy and focus (McEwen, 2007; Lupien et al., 2009). When it persists, however, it is linked to sleep disruption, mood disorders, reduced attention, and increased cardiometabolic risk (Meerlo et al., 2008; Arnsten, 2009; Steptoe and Kivimäki, 2012; Chandola et al., 2006). Such effects can affect daily functioning and worsen clinical symptoms, underscoring the need for reliable stress monitoring in healthcare and everyday life scenarios. Acutely, stress triggers sympathetic and neuroendocrine activation, temporarily heightening arousal to support attention, cognitive control, and goal-directed action (McEwen, 2007; Lupien et al., 2009). However, stress levels can shift over minutes to days and are often under-reported, meaning that single-time self-reports or sporadic biomarker measurements may miss important changes in stress level (Shiffman et al., 2008; Hellhammer et al., 2009). Additionally, for frequent or continuous stress assessment, stress monitoring should be objective and low-burden.

Conventional stress assessments rely on self-report measures, such as questionnaires and visual ratings, or peripheral physiological and chemical markers, including heart-rate variability, skin conductance, photoplethysmography (PPG), salivary cortisol, and alpha-amylase. While valuable, these approaches have notable limitations: self-reports are subjective and context-dependent, chemical assays are impractical for frequent use owing to processing delays, and PPG—though inexpensive and unobtrusive—is prone to confounds from vasomotor tone, skin temperature, respiration, posture, grip force, motion, and ambient light, which can obscure distinctions between stress and general arousal.

In contrast, neuroimaging methods can offer a direct view of brain activity related to mental stress. Functional magnetic resonance imaging (fMRI) and positron emission tomography (PET) provide high spatial resolution, but are costly, time-consuming, and confined to laboratories. More portable options include functional near-infrared spectroscopy (fNIRS) and electroencephalography (EEG). fNIRS tracks hemoglobin changes via lightweight optodes for superficial cortical mapping, yet its seconds-scale hemodynamic lag obscures rapid stress dynamics, and signals are vulnerable to systemic physiology, extracerebral flow, motion artifacts, and baseline drift. On the contrary, EEG can capture cortical electrical activity at millisecond precision, enabling extraction of stress indices in the form of oscillatory power, hemispheric asymmetry, and functional connectivity. Therefore, EEG is regarded as an ideal neuroimaging modality for detecting transient stress state changes (Berretz et al., 2022; Vanhollebeke et al., 2022; Alonso et al., 2015; Vanhollebeke et al., 2023; Paul et al., 2018; Putman et al., 2014).

For practical applications of passive brain–computer interface (BCI) outside controlled laboratory settings, traditional scalp EEG is hindered by hair interference, lengthy setup time, and sensitivity to ocular and muscular artifacts, limiting extended or frequent use. Ear-EEG addresses these issues by positioning electrodes in or around the ear (e.g., canal, concha, preauricular sites, or behind-the-ear), avoiding hair, enabling quick application, and ensuring stable contact for multi-hour recordings in daily life. However, ear-EEG shares core limitations with traditional EEG, including low single-trial signal-to-noise ratio (SNR), and susceptibility to artifacts from craniofacial muscle activity or motion (Kappel et al., 2017; Mikkelsen et al., 2015; Goverdovsky et al., 2017). Despite these limitations, research utilizing Ear-EEG is being conducted due to its advantages of wearability and applicability to wearable devices. Recent studies have demonstrated that ear-EEG supports robust data quality even in at-home or whole-night scenarios by fully leveraging the ear’s anatomy (Debener et al., 2015; Mikkelsen et al., 2019). In this study, we acquired EEG signals from bilateral preauricular points, assuming a form factor similar to commercial bone-conduction headsets (e.g., Shockz OpenRun Pro 2 and H20 Audio Tri 2 Multi-Sport Series, available in market).

To explore EEG indices associated with mental stress or to develop a stress classification model, experiments for inducing mental stress during EEG recording need to be conducted. In most previous experimental studies on mental stress, participants were assigned specific cognitive tasks designed to induce mental stress, and EEG and/or physiological signals measured during these tasks were compared with those recorded at rest (Schmidt et al., 2018; Koldijk et al., 2014; Dedovic et al., 2005; Al-Shargie et al., 2016). Examples include mental arithmetic tasks with a social-evaluative threat component (Dedovic et al., 2005; Kirschbaum et al., 1993) and protocols combining Stroop color–word interference with mental arithmetic (Al-Shargie et al., 2016). Much of this research distinguishes between task and rest periods; however, because workload and stress do not always coincide, *task-versus-rest* classification may confuse workload with stress and lead classifiers to rely on context-specific cues, reducing their generalizability across different tasks and participants (Bagheri and Power, 2020; Acharya et al., 2025). This motivates shifting the comparison away from on-task signals toward post-task rest, where residual stress can be captured without concurrent workload. Recent review articles summarized that many ML/DL pipelines rely on task-versus-rest or difficulty-based labels, so stress is entangled with workload and context (Zhou et al., 2022; Vishnu and Gupta, 2024; Kingphai and Moshfeghi, 2025). Under such settings, models may learn confounding signatures (e.g., arousal surrogates, oculomotor/EMG, display-timing) rather than stress itself.

Consistent with this concern, prior work shows that resting-state EEG can carry over from the immediately preceding task context. After learning, task-specific EEG microstates re-emerge during post-task rest, and cognitive training shifts resting-state EEG dynamics (Murphy et al., 2018; Nagy et al., 2022; Tambini and Davachi, 2013). Therefore, we compare post-stressor with post-relaxation rest to capture residual stress while minimizing concurrent task demands. Building on this basis, we implement an experimental paradigm that compares resting-state EEG recorded immediately after a stress-inducing task with that obtained after a brief meditation period. By focusing on these two rest conditions—post-stress and post-relaxation—this design avoids the conventional task–rest contrast, enabling classification models to learn features specific to mental stress rather than general markers of cognitive effort or workload. This *rest-versus-rest* protocol is expected to facilitate the isolation of stress-specific neural dynamics, thereby enabling more accurate tracking of stress fluctuations over time in practical applications. In addition, to enhance practicality for a range of passive BCI applications, we estimated users’ mental stress status in a subject-independent manner, eliminating the need for an individual calibration session prior to each system use.

Using a bilateral preauricular ear-EEG signal recorded under the rest-versus-rest paradigm, we apply deep neural networks to detect nonlinear patterns that traditional feature-based approaches may overlook. Previous studies have shown that acute stress can alter EEG activity, including changes in frontal alpha asymmetry (Berretz et al., 2022; Vanhollebeke et al., 2022) and increased functional connectivity linked to arousal and cognitive control (Alonso et al., 2015; Vanhollebeke et al., 2023; Paul et al., 2018; Putman et al., 2014). These findings suggest that deep learning architectures should explicitly model dependencies between channels. While conventional EEG classifiers such as EEGNet (Lawhern et al., 2018), ShallowConvNet, and DeepConvNet (Schirrmeister et al., 2017) work well for various BCI tasks, their basic convolution and averaging steps may not effectively capture left–right hemispherical interactions in sparse bilateral montages. To address this, we propose DeepAttNet, a channel-wise cross-attention network specifically designed for bilateral ear-EEG setting. In this architecture, each channel attends to the other channel to capture dynamic alignments related to hemispherical asymmetry, while pointwise convolutions compress features without losing band-specific energy that is important for stress detection.

Our contributions are summarized as follows:

1. Experimental paradigm: We introduce a rest-versus-rest experimental design that isolates stress-specific effects from workload by comparing two resting states—one recorded immediately after stress induction and the other after brief meditation.
2. Practical recording setup: We employ bilateral preauricular ear-EEG for enhanced applicability in practical passive BCI scenarios, and classify stress in a subject-independent manner, eliminating the need for individual calibration without per-subject normalization or tuning.
3. Model design and evaluation: We develop a deep learning architecture that combines pointwise temporal compression with channel-wise cross-attention, and benchmark it against strong baselines using eight-fold subject-level cross-validation.

## 2 Related works

### 2.1 Modulation of EEG powers and hemispheric asymmetry under mental stress

EEG offers several spectral and connectivity-based markers for detecting mental stress, reflecting brain oscillations linked to cortical arousal, emotional valence, and cognitive processing. For example, Berretz et al. (2022) used the Trier Social Stress Test to show that acute stress increases left-hemispheric activity, measured through frontal alpha asymmetry (FAA)—the log-ratio of right (AF8) to left (AF7) alpha power (8–13 Hz)—a well-known index of affective valence and approach–withdrawal tendencies. In a complementary study, Vanhollebeke et al. (2023) conducted a systematic review and meta-analysis of psychosocial stress research, finding a consistent decrease in alpha power, indicative of reduced cortical idling and increased activation, although pooled effects for beta power and FAA were non-significant due to heterogeneity across studies. In central regions, beta power (14–30 Hz) is often linked to attentional engagement, with studies such as Putman et al. (2014) and Angelidis et al. (2016) reporting stress-related increases that reflect heightened sensorimotor activity and arousal. The theta/beta ratio (theta: 4–7 Hz; beta: 14–30 Hz) represents the balance between restorative and activation rhythms; research by Clarke et al. (2019) and Putman et al. (2014) shows that imbalances in this ratio can indicate stress-related attentional deficits. High-beta coherence (23–36 Hz) between frontal and central sites is another key measure, with Alonso et al. (2015) linking it to stress-driven increases in functional connectivity. Finally, frontal midline theta (4–7 Hz) is also a robust stress marker; Cavanagh and Frank (2014) found it to rise under acute stress during tasks requiring cognitive control, reflecting increased error monitoring and sustained attention. Together, these findings motivate the introduction of new deep learning models that can effectively integrate spectral power, asymmetry, frequency ratios, and connectivity measures for improved mental stress classification (Wang et al., 2024).

### 2.2 Mental stress classification using ear-EEG

Ear-EEG has been studied for various passive BCI applications, including emotion recognition (Athavipach et al., 2019; Li et al., 2017), drowsiness detection (Nakamura et al., 2018), and cognitive workload estimation (Tremmel et al., 2024). However, its use for stress detection has been explored far less. Bae et al. (2024) recorded EEG from two ear channels and 12 scalp channels under low- and high-stress conditions. They found that high-beta power in ear-EEG and frontal alpha asymmetry in scalp-EEG could significantly distinguish stress levels. Mai et al. (2024) developed a wearable behind-the-ear EEG system with fully on-chip processing, using a small neural network to classify multiple stress levels. They later introduced (Mai and Chung, 2025) a single-channel version with integrated spectrogram conversion and a compact convolutional neural network (CNN) for stress classification. In their study, 15 participants performed stress-inducing tasks such as Stroop and mental arithmetic, and EEG signals recorded during the tasks were compared to those during rest. Across these studies, stress detection commonly relied on task-versus-rest comparisons. As mentioned above, this approach can cause models to focus on detecting task engagement rather than mental stress itself—an issue our rest-versus-rest paradigm is designed to avoid. Moreover, our deep learning method employs cross-attention mechanism to be used for bilateral preauricular ear-EEG, allowing the model to explicitly capture hemispheric asymmetries linked to mental stress, which previous simpler architectures did not address.

### 2.3 Attention-based deep learning models using multi-channel scalp-EEG

Spatial or cross-channel attention in EEG analysis mainly falls into two categories. First, some studies use attention across many scalp channels or apply Transformer-style models to image-like EEG representations. For instance, Li et al. (2024) introduced a cross-attention Swin-Transformer for cross-subject cognitive-load assessment without subject-specific training, aligning inter-domain features; Kong et al. (2025) proposed an MT-RCAF that leverages residual cross-attention for emotion recognition and mood-disorder detection. Second, other works target left–right cross-attention specifically for auditory attention decoding, such as Pahuja et al. (2023) with XAnet and Su et al. (2022) with STAnet, which jointly weight spatial channels and temporal patterns. Compared to previous research, we operate with only two preauricular ear-EEG channels under a rest-versus-rest residual-stress objective and therefore adopt a lightweight bidirectional cross-attention applied after pointwise temporal compression, avoiding global spatial pooling or image-based encodings.

## 3 Method

### 3.1 Participants

A total of 38 healthy participants with normal or corrected-to-normal vision were recruited for this study. All participants provided written informed consent prior to the experiment and received monetary compensation afterward. Before the experiment, they were informed only that the procedure involved a mental arithmetic task; it was not described as a stress-inducing task followed by stress-relief meditation. This omission was intended to minimize expectancy effects and thereby elicit genuine psychological stress during the stress-induction phase. After completing the stress-inducing task, participants were informed of the study’s true purpose and the upcoming meditation session. They then received standardized meditation instructions and proceeded to the meditation session. Data from six participants were excluded due to experimental errors, leaving 32 participants (17 males, 15 females; age range: 20–29 years) for analysis. The study protocol was reviewed and approved by the Institutional Review Board of Hanyang University (HYUIRB-202409-006-2). All participants provided written informed consent prior to participation.

### 3.2 Experiment paradigm

**Figure 1:**
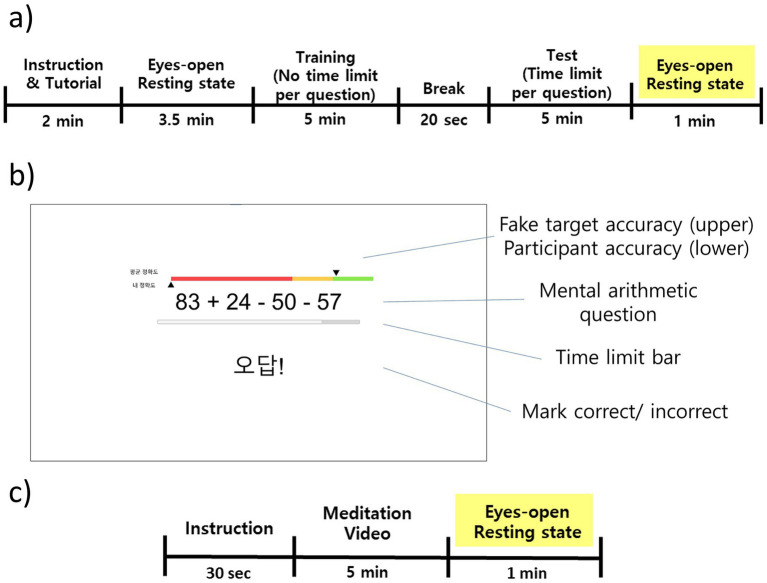
Experimental paradigms and task display. **(a)** Mental stress-inducing arithmetic protocol adapted from the Montreal Imaging Stress Task (MIST): instruction/tutorial, eyes-open resting, training block with no per-item time limit to estimate each participant’s baseline solving time, 20 s break, test block with a per-item limit set to 90% of baseline, and a 1-min eyes-open resting for the stressed-state recording. Problems used one- or two-digit operands with up to three operations; responses were entered via keypad. **(b)** Test-screen layout with sham feedback to induce social-comparison stress: a fake “80% average accuracy” indicator, under 60% target band, the arithmetic item, a time-limit bar, and correctness marking. **(c)** Stress-relief session: instruction, video-guided meditation, and a 1-min eyes-open resting.

Experimental paradigms of the mental stress-inducing task and stress-relief meditation are shown in Figure 1. The mental stress-inducing experimental paradigm was adapted from the Montreal Imaging Stress Task (Dedovic et al., 2005). It comprises two blocks of 5-min mental arithmetic using addition, subtraction, multiplication, and division. Each problem used one- or two-digit operands and up to three operations, and the correct response was a single-digit integer from 0 to 9. Participants responded by pressing the key on a provided keypad that matched the solution digit.

Before the experiment, participants completed a short practice session with a few example questions to familiarize themselves with using the keypad while maintaining visual fixation on the monitor. This was followed by a 3.5-min eyes-open resting-state recording. The first block served as a training session with no time limit, establishing each participant’s baseline per-question solving time. In the main (test) session, the time limit for each problem was set to 90% of the individual’s baseline to impose time pressure. Real-time accuracy feedback was presented alongside a target band of ≤60%, and an “80% average accuracy” indicator was prominently displayed as if it represented the participant’s mean performance to elicit social-comparison stress. This feedback was sham rather than genuine. To maintain stress levels and motivation, the time limit was adaptively adjusted: after three consecutive correct responses, it decreased by 10% for subsequent problems; after three consecutive incorrect responses, it increased by 10%.

Following the stress-inducing task, a 1-min eyes-open resting-state EEG was recorded to capture the stressed state. Immediately thereafter, a brief debriefing explained that the accuracy feedback and target band were sham elements to ensure that social-comparison stress did not persist into the relaxation phase. After the debrief, participants completed a 5-min video-guided meditation aimed at reducing stress. After the meditation session, another 1-min eyes-open resting state EEG was recorded, with posture, seating, screen distance, lighting, and fixation instructions kept identical to the earlier resting state recording.

### 3.3 Data acquisition and preprocessing

**Figure 2:**
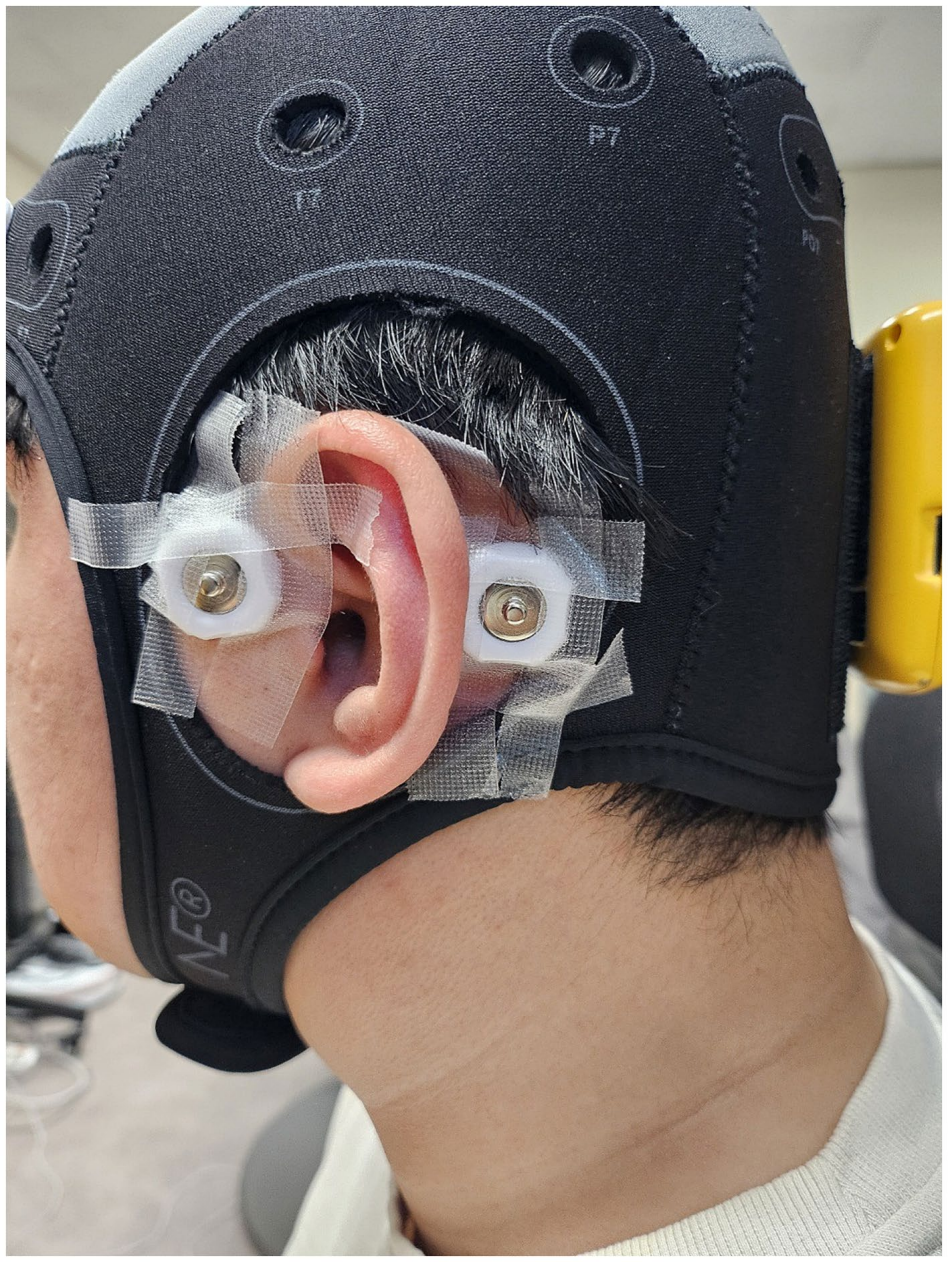
Location of a preauricular electrode positioned anterior to the left tragus. The other electrode attached to the mastoid is one of the reference electrodes.

In this study, EEG signals were recorded with an eight-channel wireless system (Enobio 8, Neuroelectrics, Barcelona, Spain) at 500 Hz using wet electrodes attached to the scalp using Signa Gel (Parker Laboratories, Fairfield, NJ, USA). We acquired data from two preauricular ear-EEG channels (Figure 2). In addition to the ear-EEG channels, six scalp-EEG electrodes (AF7, Fpz, AF8, C3, Cz, C4) were attached to the scalp to verify the effectiveness of the proposed rest-versus-rest paradigm by comparing the changes in the well-known stress-related EEG indices after the stress-inducing task and those after the meditation session. A common-mode sense active electrode and driven right-leg passive electrode were used to create a feedback loop for the amplifier reference, which was attached on left and right mastoids (see Figure 2). Ear-EEG signals were filtered with a sixth-order Butterworth bandpass filter with cutoff frequencies of 1 Hz and 40 Hz, and an additional 60 Hz notch filter was applied to reduce power-line noise. Finally, the filtered ear-EEG signals were down-sampled to 125 Hz for further analyses. For the paradigm-verification analysis, scalp-EEG was preprocessed using the same pipeline as ear-EEG.

### 3.4 Validation of experimental paradigm

To verify that our paradigm captures stress-related changes, we recorded six scalp EEG channels in addition to the two preauricular electrodes and computed seven canonical features from each 1-min post-relaxation and post-stressor segment, summarized in Table 1. First, frontal alpha asymmetry was defined as the log ratio of right AF8 to left AF7 alpha power and is widely used as an index of affective valence and approach–withdrawal (Berretz et al., 2022). Alpha power at AF7 and AF8 inversely tracks cortical arousal (Vanhollebeke et al., 2022). Beta power at Cz indexes sensorimotor and attentional engagement and typically increases under stress (Alonso et al., 2015). The theta-to-beta ratio at Cz reflects the balance between low-frequency restorative rhythms and higher-frequency arousal (Putman et al., 2014). Frontal-midline theta at Fpz relates to cognitive control and is reliably elevated under acute stress (Paul et al., 2018). Finally, high beta-band coherence between frontal and central sites captures stress-related functional coupling at the network level (Alonso et al., 2015; Vanhollebeke et al., 2023). We then compared each feature between two different resting conditions using within-subject Wilcoxon signed-rank tests, with our primary contrast defined as post-stressor versus post-relaxation. We applied formal multiple-comparison correction to guard against inflated Type I error across the seven feature-level tests.

**Table 1:** Stress-relevant EEG features used to validate the experimental paradigm.

Domain	Feature	Channel (s)	Formula or band
Power asymmetry	FAA	AF7, AF8	ln *α*(AF8) – ln *α*(AF7)
Spectral power	*α*-power	AF7	8–13 Hz
Spectral power	*α*-power	AF8	8–13 Hz
Spectral power	*β*-power	Cz	14–30 Hz
Ratio index	*θ*/*β* ratio	Cz	Power (4–7 Hz)/power (14–30 Hz)
Functional connectivity	High *β*-coherence	AF7–C3, AF8–C4, Fpz–Cz	Mean coherence at 23–36 Hz
Spectral power	Frontal-midline *θ*	Fpz	4–7 Hz

Each feature is computed per 1-min segment and summarized by its channel(s) and frequency definition. FAA (frontal alpha asymmetry) is defined as ln *α*(AF8) − ln *α*(AF7). Power denotes band power in the stated range (Hz). High-*β* coherence denotes the mean magnitude-squared coherence in 23–36 Hz across the listed frontal–central pairs.

Accordingly, we controlled the false discovery rate (FDR) using the Benjamini–Hochberg procedure. For *m* = 7 tests, let the sorted *p*-values be $p_{\left(1\right)}\leq \ldots \leq p_{\left(m\right)}$ and set *Q* = 0.05. We identified the largest *k* such that $p_{\left(k\right)}\leq (k∕m)Q$ and then declared significant all $p_{\left(i\right)}$ with i≤k . We also report the corresponding FDR-adjusted *p*-values from the same step-up procedure. Corrections were applied using standard multiple-comparison routines, yielding adjusted *p-*values and significance indicators.

### 3.5 Deep learning model architecture

**Figure 3:**
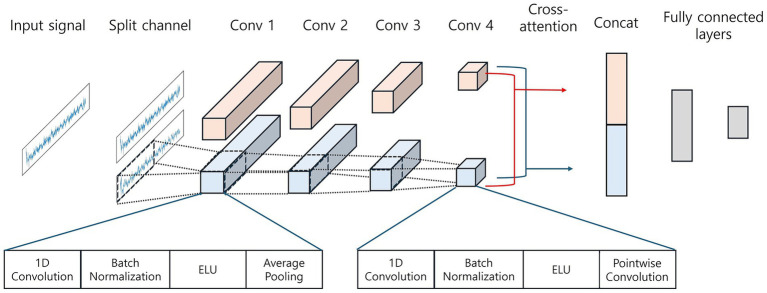
Architecture of DeepAttNet for bilateral ear-EEG stress classification. Two-channel ear-EEG (7,500 samples) is split into left and right streams. Blocks 1–3 apply 1-D convolution → batch normalization → ELU → average pooling. Block 4 applies 1-D convolution → batch normalization → ELU without pooling, followed by a 1 × 1 pointwise convolution. The per-stream feature sequences [shown as (filters × time)] enter a bidirectional cross-attention module (left → right and right → left); the attended features are concatenated and passed to fully connected layers for binary stress classification.

Motivated by prior evidence that acute stress modulates frontal alpha asymmetry as well as spectral powers, we designed a channel-wise cross-attention module to capture asymmetries between bilateral preauricular EEG channels. In our proposed DeepAttNet, as shown in Figure 3, the model takes the two preauricular ear-EEG time series (no hand-crafted features), sampled at 125 Hz after preprocessing, yielding an input tensor of (batch size, channel = 2, timestamps = 7,500) per epoch. Input data are first split into left and right channels. Each channel is processed by four consecutive temporal blocks. Each block contains a 1-D convolution kernel, batch normalization, ELU activation function, and average pooling operator. The convolution kernel lengths decrease progressively from 125 to 20 samples, which was designed to capture coarse-to-fine temporal features. A final pointwise convolution is then applied to reduce the channel dimension of each stream to a single feature map, yielding compact fixed-length embeddings that serve as queries, keys, and values. Then, to capture inter-auricular interactions, we use two cross-attention heads, originally introduced as encoder–decoder attention in the Transformer architecture for natural language processing (Vaswani et al., 2017). Cross-attention is computed bidirectionally, first with the left embedding as the query against right-side keys and values and then with the right embedding as the query against left-side keys and values, so each side conditions on the other. The attention operation is defined as:


$$\text{Attention}(Q,K,V)=\text{softmax}(\frac{QK^{⊤}}{\sqrt{d_{k}}})V\;$$

where *Q*, *K*, and *V* denote query, key, and value, respectively, $\sqrt{d_{k}}$ is the dimensionality of the key vectors used for scaling, and *T* is the number of time points in each embedding. In our implementation, $Q\in {\mathbb{R}}^{T\times d_{k}},K\in {\mathbb{R}}^{T\times d_{k}}$ , and $V\in {\mathbb{R}}^{T\times d_{v}}$ . Finally, the fused vector is passed through a shallow classifier composed of a fully connected layer, batch normalization, ELU activation, dropout, and a final linear layer to produce binary stress logits. Thus, DeepAttNet learns hierarchical temporal features, aligns them via bidirectional cross-attention between the two ear channels, and uses the combined embedding to distinguish residual stress from relaxed rest. Detailed model architecture information is referred to Supplementary 1.

### 3.6 Training details and evaluation method

We fixed all sources of randomness by seeding Python’s random, NumPy, and PyTorch with a constant seed to ensure reproducibility. Resting state epochs recorded immediately after the stress-induction task were labeled as ‘high-stress’, while those recorded after the meditation session were labeled as ‘low-stress’. We then employed an eight-fold subject-level cross-validation scheme to assess the generalization performance of the proposed model. In each fold, data from four subjects were held out for testing, and the remaining data were split into training and validation sets at a 6:1 ratio. The data were fed in mini-batches of 16 for training and eight for validation, and the full test set was evaluated in a single pass. Models were trained for up to 200 epochs, using early stopping if validation loss failed to improve for 30 consecutive epochs. Optimization was performed with the AdamW optimizer to minimize a cross-entropy loss. Hyperparameters of the model were determined through grid search. A comprehensive grid search over convolutional filter counts, kernel lengths, pooling length, pooling stride, and hidden-layer dimensions was conducted. The final hyperparameter settings are reported with the model configuration in Supplementary 1, and the main grid-search results are provided in Supplementary 2.

To benchmark the performance of DeepAttNet, we evaluated four deep neural networks widely used for EEG classification under the same training conditions. EEGNet (Lawhern et al., 2018) is a compact network model widely used in BCI research because it maintains a low parameter count by combining a single temporal convolution with depthwise spatial filtering and separable pointwise convolutions. DeepConvNet (Schirrmeister et al., 2017) extends the CNN-based EEG decoding approach by stacking four convolution–pooling blocks with small kernels and doubling the number of filters at each stage. It demonstrated strong performance in P300 detection and other standard EEG decoding tasks. ShallowConvNet (Schirrmeister et al., 2017) is a streamlined CNN-based EEG decoder. It uses two convolutional layers, one with a large temporal kernel to capture frequency-specific structure and one for spatial filtering, which makes it well suited to band-power based tasks. Finally, TSception (Ding et al., 2023) adopts an Inception (Szegedy et al., 2015)-inspired design that applies parallel temporal convolutions of multiple lengths within each block, allowing the model to learn multi-scale features; it has shown promise in cognitive workload estimation and emotion recognition. Also, to isolate and assess the contribution of cross-attention, we compare cross-attention against strong attention baselines, a self-attention variant and a lightweight Transformer (Vaswani et al., 2017), trained on the same inputs with the same protocol as proposed model. All baseline models shared the same eight-fold cross-validation, batch sizes, optimizer settings, and early-stopping criteria to ensure a fair comparison. Training configuration and computation details are described in Table 2.

**Table 2:** Training configuration and computation details used in all experiments.

Item	Value
Optimizer	AdamW (*β*₁ = 0.9, *β*₂ = 0.999, *ε* = 1e-8, amsgrad = False)
Initial learning rate	1.0 × 10^−3^ (constant; no LR scheduler)
Loss	CrossEntropyLoss (logits)
Batch size	Train: 16, validation: 8, test: full batch
Epochs/early stopping	Max 200 epochs; early stopping patience: 30
Random seed	Seed: 42 for Python/NumPy/PyTorch/CUDA
Framework/CUDA	PyTorch 2.3.1, CUDA 12.1
OS/Python	Windows 11 Educational, Python 3.9
Hardware	1 * AMD Ryzen 5 5600X, 1 * RTX 3070 Ti (VRAM 8GB)

All model evaluations use *N* = 32, eight-fold subject-level cross-validation, and 60-s segments from bilateral ear-EEG (left/right preauricular).

Performance for all models was quantified by averaging classification accuracy and macro F1-score across the eight cross-validation folds. Accuracy, defined as the proportion of correctly classified segments, summarizes overall performance. The macro F1-score—computed as the harmonic mean of precision and recall for each class and then averaged—emphasizes balanced performance across the stress and relaxation classes and penalizes asymmetric errors, providing a threshold-sensitive complement to classification accuracy.

### 3.7 Explainability analyses method of the model

To assess the explainability of the proposed model, we examined where the network places emphasis in time and across channels using cross-attention weights from the bidirectional attention module and temporal occlusion on the input time series. For parsimony and interpretability, we pre-specified two primary attention summaries: row-wise normalized entropy (concentration) and directional asymmetry (look-ahead vs. look-behind bias).

#### 3.7.1 Cross-attention weights

During inference, we recorded the attention tensors from the two cross-attention blocks (left → right, right ← left). For each sample, the attention has shape T′×T′ ( T′=35) , where rows index query time bins and columns index key time bins. We grouped samples by label (post-stress vs. post-relaxation) and computed label-wise means within each fold.

*Primary index 1*: Row-wise normalized entropy. Let $A\in {\mathbb{R}}^{T\prime \times T\prime }$ denote the cross-attention matrix for one direction, where $A_{\mathrm{ij}}$ is the weight assigned by the query at time bin i to the key at time bin j . for each row i , we form $P_{i,j}$ and row entropy $H_{i}$ as:


$$P_{i,j}=\frac{A_{i,j}}{\sum_{j}A_{i,j}},H_{i}=-\sum_{\dot{j}}P_{i,j}\log P_{i,j}$$

Lower values indicate sharper, more concentrated attention, and higher values indicate more uniform spread.

*Primary index 2*: Directional asymmetry. We quantify non-symmetry as:


$$\frac{{\left\|A-A^{⊤}\right\|}_{F}}{{\left\|A\right\|}_{F}}$$

where F stands for Frobenius norm. Higher values indicate stronger directional bias, whereas 0 indicates a symmetric map.

#### 3.7.2 Temporal occlusion

To assess the contribution of local time segments, we slid a zero-baseline window over the two-channel input and measured the change in class logit. With a window width W=0.5s and step 0.1s , both channels were zeroed within the window. For class c∈{Stress,Relax} , we computed:


$${\text{Δlogit}}_{c}(t)={\text{logit}}_{c}(x)-{\text{logit}}_{c}(x_{\mathrm{occ}}(t))$$

Positive values indicate supportive segments for class c since masking reduces the logit, and negative values indicate counterevidence. Within each fold we averaged $\Delta {\text{logit}}_{c}(t)$ over samples of the same label and then averaged across folds. For reporting we also extracted the time of the maximum $\Delta {\text{logit}}_{c}$ and the signed area of positive and negative parts.

## 4 Results

### 4.1 Feature-level comparison of post-stress versus post-relaxation resting state

**Figure 4:**
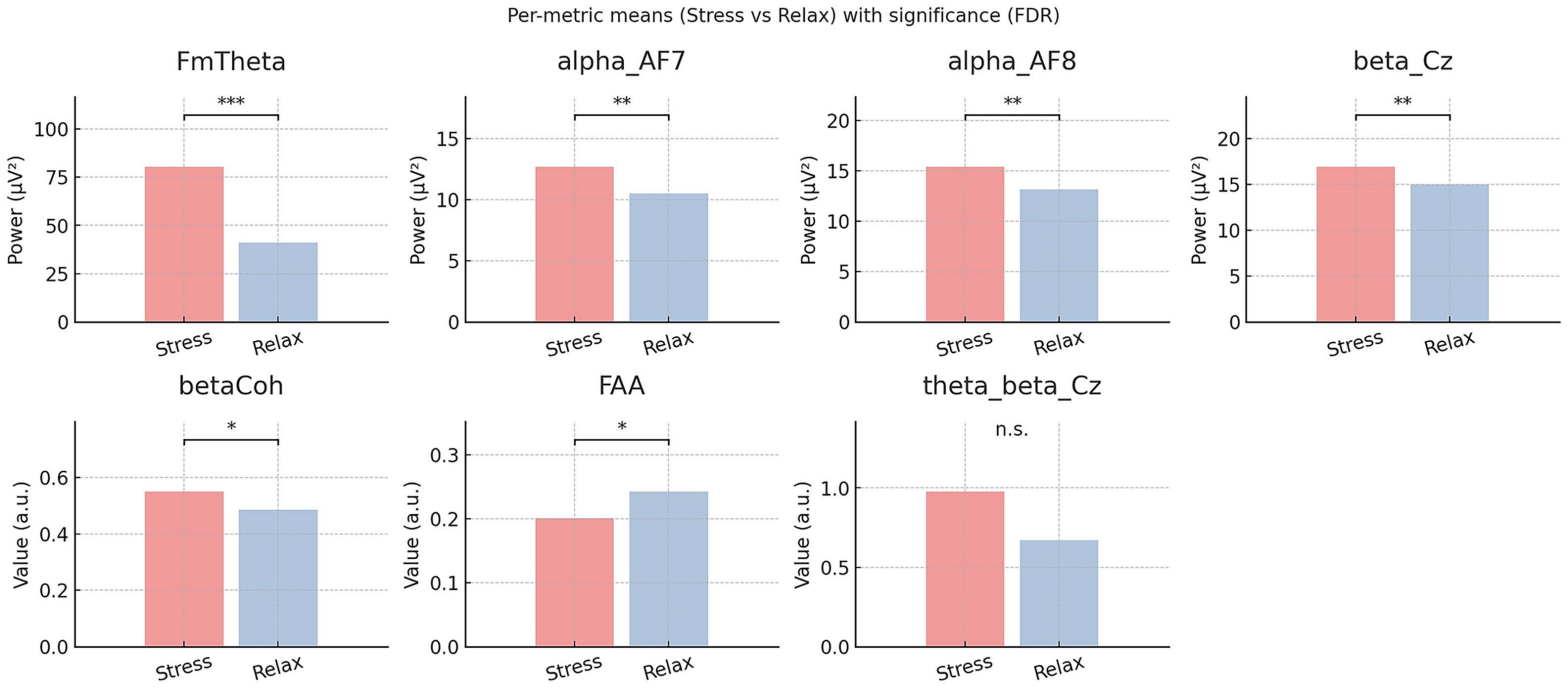
Stress–relax comparison of canonical EEG features computed from 60-s eyes-open resting segments. Bars show subject-paired means for each metric: frontal-midline theta power (FmTheta), α-power at AF7/AF8, *β*-power at Cz, high *β*-coherence (betaCoh), frontal alpha asymmetry (FAA) and Cz *θ*/*β* ratio. Power features are in μV^2^; others are in a.u. Colors: Stress (light red), Relax (light blue). Significance was tested with two-sided Wilcoxon signed-rank across subjects and Benjamini–Hochberg FDR correction; brackets mark significant contrasts (*, **, and *** represent *q* values less than 0.05, 0.01, and 0.001, respectively; n.s., not significant). Significant differences were observed for FmTheta (***), AF7 *α* (**), AF8 *α* (**), Cz *β* (**), *β*-coherence (*), and FAA (*). Cz θ/*β* was n.s.

This analysis tested whether the proposed experimental paradigm could capture mental stress changes by comparing canonical EEG markers between the post-stress and post-relaxation resting states. We computed seven stress-relevant features using six scalp EEG channels for each 1-min segment (see Table 1) and compared the EEG feature values between two resting states using within-subject Wilcoxon signed-rank tests. *p*-values were controlled for multiple testing using FDR correction at *Q* = 0.05. Across the stress versus relax contrast, frontal-midline theta, high *β*-band coherence, Cz *β* power, AF7 *α* power, and AF8 *α* power remained significant after FDR correction, whereas only the *θ*/*β* ratio at Cz did not. Table 3 reports unadjusted and FDR-corrected p-values for the primary contrast, alongside Rosenthal’s r (*r* = *|Z*|/√*n*), with FDR-significant differences highlighted in Figure 4. These results, showing significant changes in six of seven representative stress-related EEG features, validate the rest-versus-rest paradigm’s effectiveness in isolating stress-specific neural dynamics. Note that these seven hand-crafted scalp-EEG features were computed *post hoc* for paradigm validation and were not used as inputs to deep learning models.

**Table 3:** Within-subject Wilcoxon signed-rank comparison of post-stressor versus post-relaxation rest for seven EEG markers.

Feature	*p-*value	*q*-value	Rosenthal’s *r*
	Without correction	FDR-corrected	
FAA	0.0280 (*)	0.0367 (*)	0.387
AF7 *α*-power	0.0016 (**)	0.0054 (**)	0.543
AF8 *α*-power	0.0026 (**)	0.0054 (**)	0.519
Cz *β*-power	0.0054 (**)	0.0098 (**)	0.483
Cz *θ*/*β* ratio	0.1901	0.2348	0.235
High *β*-coherence	0.0228 (*)	0.0319 (*)	0.284
Frontal–midline *θ*	<2.00e-8 (***)	<4.20e-7 (***)	0.840

Table reports unadjusted *p-*values and FDR-adjusted *q-*values. The rightmost column gives the Rosenthal’s *r* with the sign reflecting the median of stress–relax. Significant results after FDR control (*q* < 0.05) are highlighted. *, **, and *** represent *p* (or *q*) values less than 0.05, 0.01, and 0.001, respectively.

### 4.2 Subject-independent classification performance

#### 4.2.1 Comparison with conventional deep learning models

Table 4 reports the mean ± standard deviation of accuracy and macro F1-score across the eight subject-level cross-validation folds. As shown in the table, the proposed DeepAttNet outperformed all baselines, achieving the highest accuracy and macro F1-score. Compared to the next-best baseline, DeepConvNet, DeepAttNet shows substantial improvements in both metrics. Similar gains are observed relative to the other models. The consistent ranking across both metrics suggests that these improvements stem from a balanced enhancement in precision and recall, rather than favoring one class over the other. To assess statistical significance, we compared DeepAttNet with each baseline using the Wilcoxon signed-rank test across the eight folds and applied Benjamini–Hochberg FDR correction. Statistical results are referred to Supplementary 3.

**Table 4:** Average accuracy and macro F1-score of eight-fold subject-level cross-validation for binary mental stress level classification with 60-s bilateral preauricular ear-EEG segments input.

Model	Accuracy (%)	Macro F1-score
ShallowConvNet	65.63 ± 10.36	0.6332 ± 0.1141
DeepConvNet	67.19 ± 6.05	0.6501 ± 0.0722
EEGNet	59.36 ± 14.99	0.5762 ± 0.1569
TSception	64.06 ± 11.59	0.6295 ± 0.1246
**DeepAttNet (proposed)**	**76.56 ± 4.42**	**0.7612 ± 0.0458**

Bold represents best performance compared to other models.

#### 4.2.2 Ablation study

We conducted an ablation study to isolate and quantify the contribution of the two key components in DeepAttNet: channel-wise cross-attention and pointwise temporal convolution. Four variants were evaluated under the same eight-fold subject-level cross-validation: the proposed model, a model without cross-attention, a model without pointwise convolution, and a model without both.

**Figure 5:**
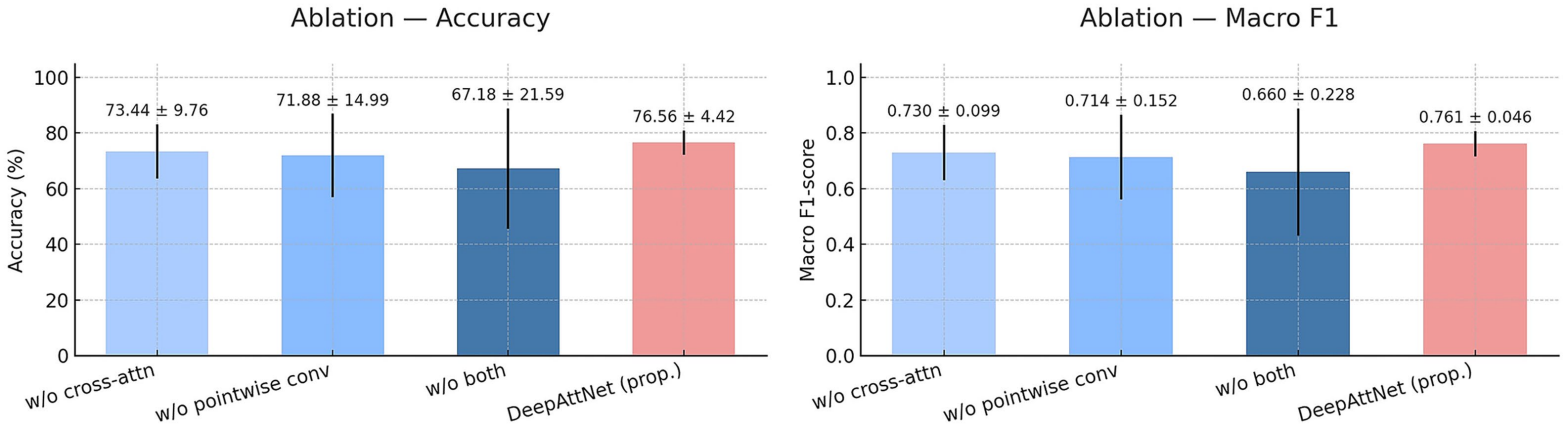
Ablation study of DeepAttNet components on ear-EEG stress classification with 60-s bilateral ear-EEG segments. Left: accuracy; right: macro F1-score. Bars show eight-fold means with ±SD error bars, and numeric labels denote mean ± SD. Models include w/o cross-attention, w/o pointwise conv, w/o both, and DeepAttNet. The proposed model achieves the best performance across both metrics.

As summarized in Figure 5, the proposed model achieved the highest accuracy and macro F1-score across folds. Removing cross-attention reduced both metrics, and removing pointwise convolution also led to a measurable decrease. The model with both variants absent yielded the lowest performance among the four. The same ordering was observed for both accuracy and macro F1-score, indicating a consistent degradation when either component is omitted. The exact numerical values are provided in Supplementary 4.

To isolate the contribution of cross-attention, we implemented two baselines that consume the same encoder outputs and inputs. The first is a self-attention variant that applies within-channel multi-head self-attention followed by temporal averaging. The second is a lightweight Transformer that concatenates left/right token streams, adds positional and channel embeddings, and applies a single-layer Transformer encoder with two heads. Both baselines were trained with the same optimizer, schedule, and regularization. Table 5 reports parameter counts, FLOPs (computational cost), and the mean ± SD of accuracy and macro F1-score across eight folds. Across the attention-based modules, cross-attention achieves the highest accuracy and macro F1-score, while remaining within a most compact parameter/FLOP budget.

**Table 5:** Ablation of attention mechanisms in DeepAttNet for bilateral preauricular ear-EEG stress classification with 60-s resting state segments.

Module	Params	FLOPS	Heads/layers	Accuracy (%)	Macro F1-score
Self-attention	0.25 M	77.81 M	1/1	71.88 ± 14.99	0.6954 ± 0.1867
Lightweight transformer	**0.26 M**	**78.25 M**	2/1	68.75 ± 10.83	0.6809 ± 0.1165
**Cross-attention (proposed)**	**0.07 M**	**71.21 M**	1/2^a^	**76.56 ± 4.42**	**0.7612 ± 0.0458**

a Two directional cross-attention modules (left → right and right → left).

We compare self-attention and a lightweight transformer to the proposed cross-attention under the same inputs and training protocol. Bold indicates the best performance among models.

### 4.3 Model explainability of the proposed model

#### 4.3.1 Cross-attention weights

**Figure 6:**
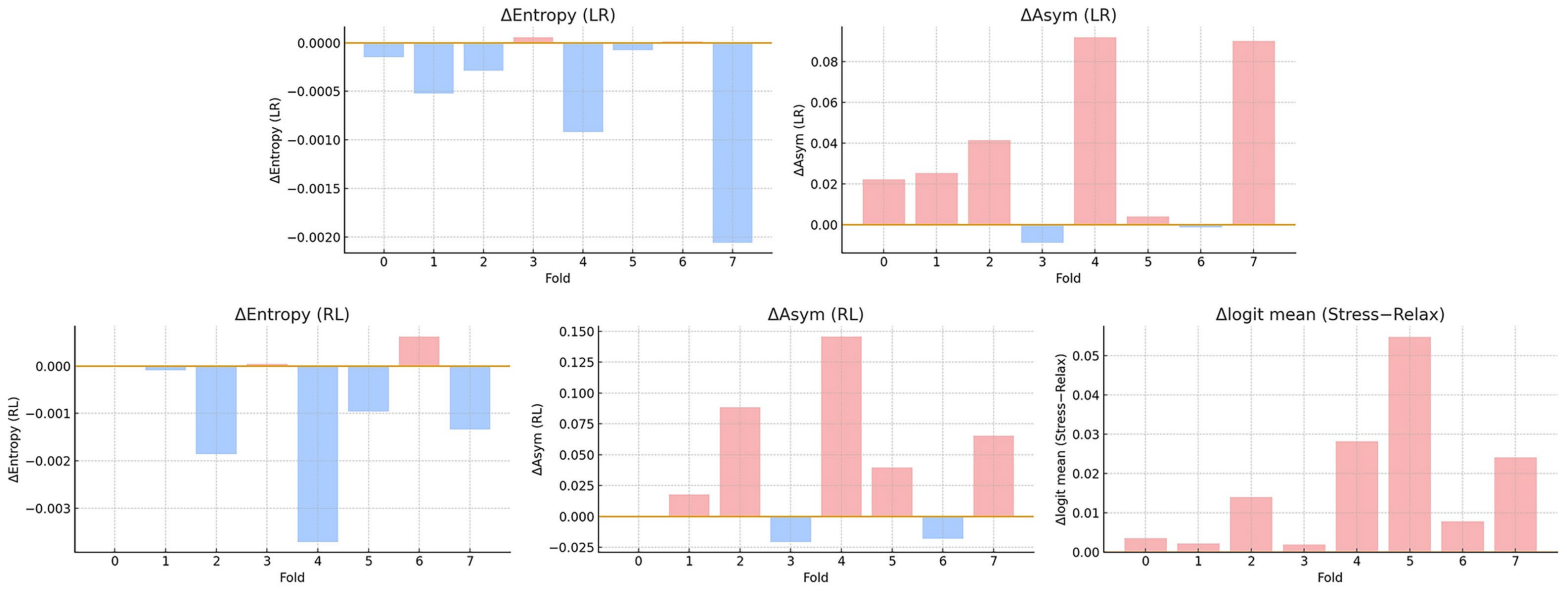
Per-fold deltas (stress−relax) for attention and occlusion. Top: *Δ* Entropy (left–right; LR), *Δ* Asymmetry (LR). Bottom: *Δ* Entropy (right–left; RL), *Δ* Asymmetry (RL), and the mean Δlogit from temporal occlusion. Bars are colored light red for positive and light blue for negative values; the horizontal line marks zero. Negative *Δ* Entropy indicates more concentrated attention under stress, positive Δ Asymmetry indicates stronger directional imbalance, and positive mean *Δ* logit indicates that masking removes more evidence for stress than for relax.

Using the per-fold deltas in Figure 6, we observe a consistent but small shift under stress. For ΔEntropy (stress−relaxation), six of the eight folds are negative in the LR direction and five of the eight in the right–left direction (two right–left folds are near-zero positives). The across-fold mean ΔEntropy is −4.9 × 10^−4^ in left–right and −9.1 × 10^−4^ in right–left, indicating slightly more concentrated attention under stress. For ΔAsymmetry , six folds in left–right and five folds in right–left are positive, with across-fold means 0.033 (left–right) and 0.040 (right–left).

**Figure 7:**
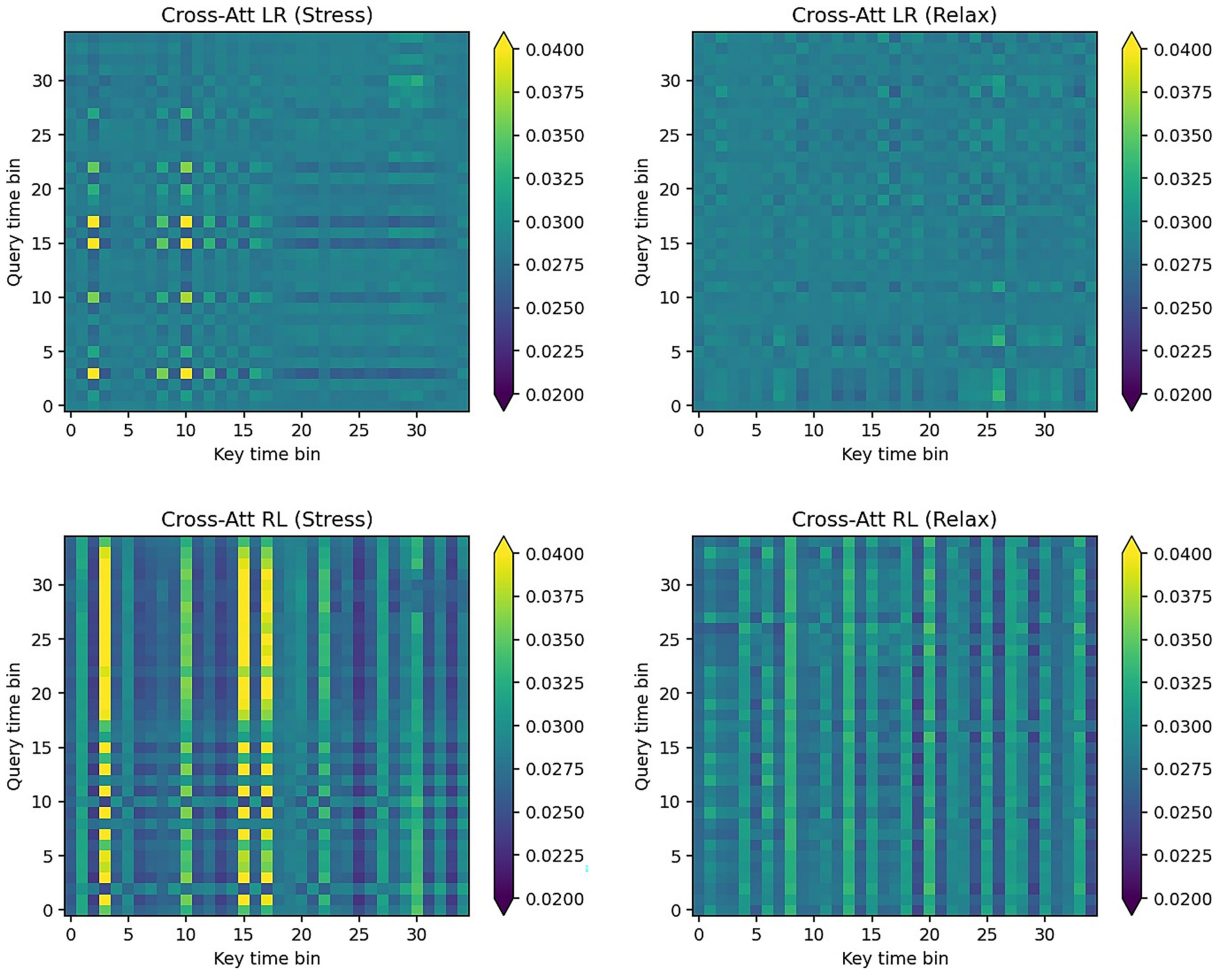
Representative cross-attention maps of single fold. Axes show key (*x*) and query (*y*) time bins. A common color scale is used across panels for comparability. Relative to relax, stress exhibits moderately sharper and more directional patterns at several time bins.

Overall, stress tends to show lower entropy, meaning more concentrated distributions, and higher directional asymmetry than relaxation. Figure 7 is an illustrative example of cross-attention maps.

#### 4.3.2 Temporal occlusion

**Figure 8:**
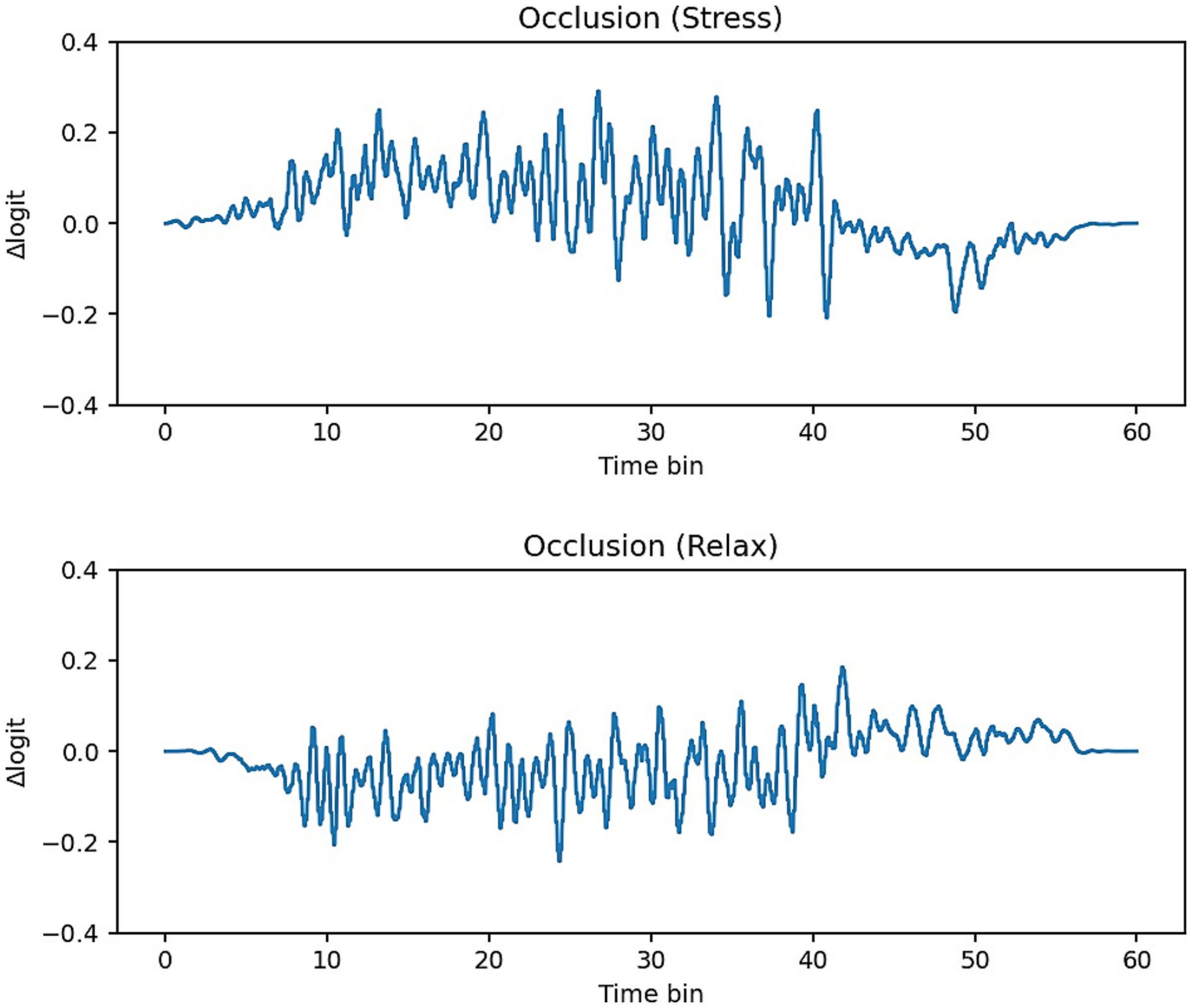
Temporal occlusion curves (Δlogit per time bin) for a representative fold. Positive Δlogit denotes time segments whose masking reduces class evidence (supportive regions). Stress shows larger positive mass and mid–late peaks, whereas relax contains more negative segments (counterevidence). The same *y*-axis range is used for both panels.

As shown in Figure 6, sliding-window occlusion revealed larger positive Δlogit for stress than relax in every fold, indicating more extensive supportive time segments for the stress class. The effect was clearest in fold 5 and fold 4 (Figure 8). In fold 5, stress showed a higher mean Δlogit (≈ + 0.0348) while Relax was negative (≈ − 0.0200), with a large positive-mass area (stress ≈ 396, relax ≈ 119). Overall, Stress concentrates modestly more attention and contains mask-sensitive supportive segments in mid–late windows, whereas Relax exhibits more counterevidence (negative Δlogit ) segments. We note that attention-side effects are small in magnitude, and fold-to-fold variability persists; we therefore report them as trends rather than strong mechanistic claims.

## 5 Discussion

Performance of subject-independent classification of mental stress was improved when a new deep learning model incorporated with a cross-attention mechanism was applied to bilateral ear-EEG data. The proposed model architecture enabled each channel to explicitly condition its representation on the other, thereby enhancing the model’s ability to capture inter-auricular dependencies that may be underutilized in conventional convolutional operations. As a result, across all tested architectures, DeepAttNet achieved the highest accuracy and macro-F1 scores. Ablation analyses further confirmed the contribution of the proposed components: removal of either the cross-attention module or the pointwise temporal compression resulted in performance decrements. These results suggest that both modules played complementary roles in extracting discriminative features from sparse bilateral channel configurations—cross-attention by modeling hemispheric asymmetries and temporal coordination, and pointwise compression by preserving band-specific energy while reducing dimensionality. As an additional ablation, we replaced the cross-attention block with two alternatives—channel-wise self-attention and lightweight transformer. All variants improved over the no-attention setting but still underperformed the original cross-attention model in both accuracy and macro-F1. This suggests that the main gain comes from modeling ear-to-ear (inter-auricular) interactions in a directional way, where each ear conditions its representation on the other. By contrast, modules that only reweigh features within a channel or treat channels without direction captured less of this dependency. The adoption of a rest-versus-rest protocol likely further enhanced generalization by minimizing the influence of workload differences and stimulus context on the decision boundary, allowing the classifier to focus more specifically on stress-related features.

Beyond performance, our explainability analyses suggest that the cross-attention module exploits bilateral (left–right) interactions in ear-EEG. This interpretation aligns with temporal EEG evidence. At rest, high-frequency (23–36 Hz) T3/T4 asymmetry shows that rightward dominance is associated with higher resting heart rate and lower baroreflex sensitivity, consistent with sympathetic predominance, whereas leftward dominance aligns with parasympathetic/vagal influences (Tegeler et al., 2015). In a case series, movement toward symmetry was accompanied by increases in HRV (SDNN) and BRS, and baseline rightward asymmetry was negatively correlated with HRV (Tegeler et al., 2017). Accordingly, bilateral ear-EEG, which samples near the temporal regions, may capture interaural temporal asymmetries. Our per-fold attention summaries and temporal-occlusion responses are consistent with this account, although effect sizes are small and variable. We therefore present this as a plausible physiological rationale rather than dispositive evidence.

Before applying our method to EEG classification, we investigated whether the proposed rest-versus-rest protocol could reliably detect changes in mental stress. To this aim, we analyzed well-known stress-related EEG markers from multiple scalp locations, even though these signals were not used in the classification model. The results showed clear differences between the two rest periods after FDR correction. The strongest effect was an increase in frontal midline theta at Fpz. We also found that high beta-band coherence between frontal and central sites reliably separated the two resting states. In addition, central beta power increased after the stress task, and frontal alpha asymmetry shifted toward lower asymmetry. Together, these findings suggest that the proposed protocol created a meaningful physiological difference between the post-stressor and post-relaxation rest periods. These patterns match previous findings showing that frontal midline theta rises with higher cognitive control demands, and that beta-band connectivity reflects coordinated engagement after stress (Berretz et al., 2022; Vanhollebeke et al., 2022; Alonso et al., 2015; Vanhollebeke et al., 2023; Paul et al., 2018; Putman et al., 2014).

Several limitations of the present study need to be acknowledged. First, the sample size was relatively small and drawn from a single site, which may limit the generalizability of the findings to broader and more diverse populations. Second, spatial coverage was restricted to a sparse two-channel preauricular montage. Although this configuration was selected for its simplicity and potential suitability in wearable applications, it inevitably constrains the ability to capture neural dynamics from more distal or distributed cortical regions. Third, wet electrodes were used to ensure low-impedance contact and high signal quality; however, their need for conductive gel and cleaning reduces convenience for everyday use. For real-world deployment, lower-maintenance alternatives such as dry or semi-dry electrodes may be preferable, even if some signal fidelity is sacrificed. Also, the current study is limited by its sample of 32 young adults, which may constrain generalizability to broader populations.

While scalp EEG provided neural validation of the rest-versus-rest paradigm, our 60-s resting blocks were generally insufficient for robust heart rate variability indices, which typically require longer windows (2–5 min, Shaffer and Ginsberg, 2017; Sarmiento et al., 2013) for reliable frequency-domain analysis, and incompatible with on-session hormonal sampling (e.g., salivary cortisol), which involves post-event delays of 15–20 min for peak responses (Kudielka et al., 2009; Ali and Pruessner, 2012). As a result, the physiological interpretability of EEG findings is limited without convergent evidence from peripheral measures.

These limitations suggest several avenues for future research. Expanding the ear electrode array or pairing ear-EEG with a minimal scalp set could enhance connectivity estimates while maintaining wearability. Profiling on-device inference latency and energy consumption will be essential for continuous, real-world monitoring of mental stress. Replication with independent datasets and prospective ambulatory studies will further establish generalizability. We did not use data augmentation in this study; however, prior work shows that channel-level recombination can improve EEG classification (Pei et al., 2021). Replicating with larger, more diverse cohorts across age, ethnicity, and clinical subgroups would strengthen external validity. Additionally, incorporating multimodal signals, such as pairing EEG with PPG-based heart rate variability and electrodermal activity, could provide physiological evidence to enhance validation of stress-related responses; where feasible, noninvasive hormonal assays (e.g., salivary cortisol) should be considered to further substantiate the paradigm’s physiological grounding. Future studies should also include objective physiological markers and brief self-reports to verify the intended state. Lastly, to further reduce context sensitivity, future works should include negative-control contrasts (e.g., high-workload/low-stress), and record nuisance channels for electrooculography (EOG) and EMG regression.

## 6 Conclusion

In this study, mental stress of a user could be classified with a high classification accuracy in a subject-independent manner using only two-channel bilateral ear-EEG. Unlike the previous studies, this study proposed a rest-versus-rest protocol to isolate mental stress from other cognitive processes. Our proposed DeepAttNet, designed to capture inter-auricular dynamics through cross-attention and pointwise temporal compression, consistently outperformed traditional EEG classifiers, with ablation results highlighting that both added modules were critical. Also, by model explainability analysis, model showed consistent with the intended use of cross-attention to capture ear-to-ear dependencies in two-channel recordings. These results not only establish bilateral ear-EEG as a viable platform for passive stress monitoring, but also mark a decisive step toward bringing continuous, brain-based stress sensing out of the lab and into everyday life.

## Data Availability

The raw and processed EEG datasets will be made available by the authors upon reasonable request. De-identified data sufficient to reproduce the main figures will be deposited in a public repository before publication. Training and evaluation code along with model structure code of DeepAttNet is available in a Github repository: https://github.com/WsHyung/DeepAttNet/tree/main.
