# Supplementary material for: DeepAttNet: deep neural network incorporating cross-attention mechanism for subject-independent mental stress detection in passive brain–computer interfaces using bilateral ear-EEG
Source: Front Hum Neurosci. 2025 Nov 3;19:1685087. doi: 10.3389/fnhum.2025.1685087 (PMC12620360; doi:10.3389/fnhum.2025.1685087)
Supplement: Supplementary file 1 [file Table_1.DOCX]

Supplementary Material

**Supplementary 1.**

Supplementary Table S1. Layer-by-layer breakdown of DeepAttNet

| **Layer** | **Type** | **Kernel Size / Parameters** | **Stride / Padding** | **Output Shape** | **Activation** |
| --- | --- | --- | --- | --- | --- |
| Input | Input | N/A | N/A | (B, 1, 2, 7500) | N/A |
| Channel Split (Left/Right) | Slice | N/A | N/A | (B, 1, 1, 7500) per channel | N/A |
| Temporal Conv 1 (per channel) | Conv2d | (1, 125), out_channels=16 | (1, 1) / (0, 0) | (B, 16, 1, 7376) | None |
| BatchNorm 1 (per channel) | BatchNorm2d | N/A | N/A | (B, 16, 1, 7376) | None |
| Activation 1 (per channel) | ELU | N/A | N/A | (B, 16, 1, 7376) | ELU |
| Pool 1 (per channel) | AvgPool2d | (1, 5) | (1, 5) | (B, 16, 1, 1475) | None |
| Temporal Conv 2 (per channel) | Conv2d | (1, 20), out_channels=32 | (1, 1) / (0, 0) | (B, 32, 1, 1456) | None |
| BatchNorm 2 (per channel) | BatchNorm2d | N/A | N/A | (B, 32, 1, 1456) | None |
| Activation 2 (per channel) | ELU | N/A | N/A | (B, 32, 1, 1456) | ELU |
| Pool 2 (per channel) | AvgPool2d | (1, 5) | (1, 5) | (B, 32, 1, 291) | None |
| Temporal Conv 3 (per channel) | Conv2d | (1, 20), out_channels=32 | (1, 1) / (0, 0) | (B, 32, 1, 272) | None |
| BatchNorm 3 (per channel) | BatchNorm2d | N/A | N/A | (B, 32, 1, 272) | None |
| Activation 3 (per channel) | ELU | N/A | N/A | (B, 32, 1, 272) | ELU |
| Pool 3 (per channel) | AvgPool2d | (1, 5) | (1, 5) | (B, 32, 1, 54) | None |
| Temporal Conv 4 (per channel) | Conv2d | (1, 20), out_channels=128 | (1, 1) / (0, 0) | (B, 128, 1, 35) | None |
| BatchNorm 4 (per channel) | BatchNorm2d | N/A | N/A | (B, 128, 1, 35) | None |
| Activation 4 (per channel) | ELU | N/A | N/A | (B, 128, 1, 35) | ELU |
| Dimension Reduction (per channel) | Conv2d | (1, 1), out_channels=1 | (1, 1) / (0, 0) | (B, 1, 1, 35) | None |
| Reshape (per channel) | View | N/A | N/A | (B, 35, 1) | N/A |
| Cross-Attention (L to R) | CrossAttention | embed_dim=35 | N/A | (B, 35, 1) | Softmax (weights) |
| Cross-Attention (R to L) | CrossAttention | embed_dim=35 | N/A | (B, 35, 1) | Softmax (weights) |
| Feature Concatenation | Cat | dim=1 | N/A | (B, 70) | N/A |
| Dense 1 | Linear | in_features=70, out_features=64 | N/A | (B, 64) | None |
| BatchNorm Dense | BatchNorm1d | N/A | N/A | (B, 64) | None |
| Activation Dense | ELU | N/A | N/A | (B, 64) | ELU |
| Dropout | Dropout | p=0.3 | N/A | (B, 64) | N/A |
| Output Layer | Linear | in_features=64, out_features=2 | N/A | (B, 2) | None |

**Supplementary 2.**

Main hyperparameter grid search results for DeepAttNet.
For conciseness, we decompose the search into four parts while fixing the remaining hyperparameters to their optimal values: (i) number of filters and length of filter for initial temporal convolution, (ii) pooling length and stride for three temporal pooling filters, (iii) number of filters for temporal convolution 2, 3, 4, and (iv) number of the hidden node for classifier. For each part, only the listed hyperparameters vary, and we report mean ± SD accuracy and macro F1 across eight folds under the same training protocol.

Table S2a. Initial temporal-convolution sweep.

Grid over number of filters and length of filter in initial temporal convolution for DeepAttNet on ear-EEG stress classification. All other hyperparameters are fixed to their optimal settings. Results are reported as mean ± SD accuracy and macro F1 over 8-fold CV with identical data splits, optimizer, and schedule. Bold represents best performance.

| **Filter counts** | **Filter length** | **Accuracy (%)** | **Macro F1-Score** |
| --- | --- | --- | --- |
| **16** | **125** | **76.56 ± 4.13** | **0.7612 ± 0.0428** |
| 32 | 125 | 70.31 ± 10.71 | 0.6743 ± 0.1492 |
| 64 | 125 | 67.19 ± 10.71 | 0.6184 ±0.1746 |
| 32 | 62 | 64.06 ± 11.59 | 0.6017 ±0.1483 |
| 64 | 62 | 64.06 ± 9.76 | 0.5884 ±0.1369 |
| 16 | 62 | 56.25 ± 15.31 | 0.5043 ±0.1989 |

Table S2b. Temporal pooling sweep.

Grid over temporal pooling length and stride for DeepAttNet on ear-EEG stress classification. All other hyperparameters are fixed to their optimal settings. Results are reported as mean ± SD accuracy and macro F1 over 8-fold CV with identical data splits, optimizer, and schedule. Bold represents best performance.

| **Pooling length** | **Pooling stride** | **Accuracy (%)** | **Macro F1-Score** |
| --- | --- | --- | --- |
| **5** | **5** | **76.56 ± 4.13** | **0.7612 ± 0.0428** |
| 10 | 5 | 64.06 ± 13.17 | 0.6028 ± 0.1688 |
| 5 | 2 | 64.06 ± 14.57 | 0.5529 ± 0.2234 |
| 10 | 2 | 56.25 ± 8.84 | 0.4568 ± 0.1639 |

Table S2c. Downstream filter-count sweep.

Grid over downstream temporal convolution filter counts for DeepAttNet on ear-EEG stress classification. All other hyperparameters are fixed to their optimal settings. Results are reported as mean ± SD accuracy and macro F1 over 8-fold CV with identical data splits, optimizer, and schedule. Bold represents best performance.

| **Filter counts for temporal conv. 2** | **Filter counts for temporal conv. 3** | **Filter counts for temporal conv. 4** | **Accuracy (%)** | **Macro F1-Score** |
| --- | --- | --- | --- | --- |
| **32** | **32** | **128** | **76.56 ± 4.13** | **0.7612 ± 0.0428** |
| 32 | 64 | 128 | 71.88 ± 12.10 | 0.6938 ± 0.1633 |
| 16 | 32 | 128 | 62.50 ± 10.83 | 0.5735 ± 0.1632 |
| 16 | 64 | 64 | 62.50 ± 13.98 | 0.5677 ± 0.2021 |
| 32 | 32 | 64 | 60.94 ± 14.57 | 0.5385 ± 0.1998 |
| 32 | 64 | 64 | 59.38 ± 8.27 | 0.5213 ± 0.1536 |
| 16 | 32 | 64 | 59.38 ± 10.36 | 0.5020 ± 0.1783 |
| 16 | 64 | 128 | 76.56 ± 8.84 | 0.4943 ± 0.1479 |

Table S2d. Classifier width sweep.

Grid over classifier hidden size for DeepAttNet on ear-EEG stress classification. All other hyperparameters are fixed to their optimal settings. Results are reported as mean ± SD accuracy and macro F1 over 8-fold CV with identical data splits, optimizer, and schedule. Bold represents best performance.

| **Hidden size** | **Accuracy (%)** | **Macro F1-Score** |
| --- | --- | --- |
| **64** | **76.56 ± 4.13** | **0.7612 ± 0.0428** |
| 128 | 64.06 ± 14.57 | 0.6191 ± 0.1724 |

**Supplementary 3.**

DeepAttNet showed significant improvements over EEGNet for both accuracy and macro F1.

Against ShallowNet, DeepAttNet was significant for macro and showed a positive trend for accuracy. Relative to DeepConvNet, the difference favored DeepAttNet with a near-significant effect in macro F1 and a positive trend in accuracy. Differences verses TSception were not significant after FDR correction. Overall, these non-parametric tests corroborate that the proposed model’s gains—especially in macro F1—are robust across folds under FDR control.

Supplementary Table S3. Wilcoxon signed-rank tests comparing DeepAttNet with each baseline across 8 folds. Values report the difference between DeepAttNet verses baseline models. P denotes the two-sided Wilcoxon p-value, and q(FDR) applies Benjamini–Hochberg correction across all tests. Additionally, Rosenthal’s r is reported. * represent p (or q) values less than 0.05.

| **Feature** | **Models (vs DeepAttNet)** | ***p-*value** | ***q*-value** | | **Rosenthal’s *r*** |
| --- | --- | --- | --- | --- | --- |
|  |  | **W/o correction** | | **FDR-corrected** |  |
| **Accuracy** | ShallowConvNet | 0.094 | | 0.125 | 0.752 |
|  | DeepConvNet | 0.031 (*) | | 0.083 | 0.797 |
|  | EEGNet | 0.063 | | 0.125 | 0.890 |
|  | TSception | 0.313 | | 0.313 | 0.640 |
| **Macro  F1-Score** | ShallowConvNet | 0.078 | | 0.125 | 0.683 |
|  | DeepConvNet | 0.016(*) | | 0.083 | 0.831 |
|  | EEGNet | 0.031 (*) | | 0.083 | 0.878 |
|  | TSception | 0.281 | | 0.313 | 0.439 |

**Supplementary 4.**

Supplementary Table S4. Main ablation results for DeepAttNet on ear-EEG stress classification. Values are 8-fold mean ± SD (Accuracy in %, Macro F1 unitless). Variants include removal of cross-attention, pointwise convolution, or both; all models share identical encoder, training protocol, and data splits. Bold denotes the best performance in each column.

| **Model** | **Accuracy (%)** | **Macro F1-Score** |
| --- | --- | --- |
| w/o cross-attention | 73.44 ± 9.76 | 0.7296 ± 0.0992 |
| w/o pointwise convolution | 71.88 ± 14.99 | 0.7142 ± 0.1523 |
| w/o cross-attention & pointwise convolution | 67.18 ± 21.59 | 0.6597 ± 0.2281 |
| **DeepAttNet (Proposed)** | **76.56 ± 4.42** | **0.7612 ± 0.0458** |
